# Congenital Absence of the Patellæ—Stigma of Degeneracy

**Published:** 1898-01

**Authors:** 


### CONGENITAL ABSENCE OF THE PATELLÆ—STIGMA OF DEGENERACY.

H. N. Moyer contributes to *The Lancet* of November 27, 1897, the description of a patient with congenital absence of both patellæ, presenting extraordinary signs of degeneracy. His head, which was large, was decidedly asymmetrical, the left half of the face being somewhat larger than the right. The skull was exceedingly irregular in its osseous conformation. Both ears were very small and irregular and the ordinary oracular markings not very apparent. There was microphthalmus of the right eye with apparently congenital haziness of the cornea. Most of the teeth were lost, but the dental arches were well formed. The arm and forearm were fairly well-developed, but each thumb was broad and both thumb-nails had a central groove. There was a rudimentary finger on each hand. Over the lower end of the sacrum was a fold of skin which rose somewhat above the surrounding level and concealed a slight opening in which a probe could be passed for a depth of about one-fourth of an inch. The testicles were undescended. The thighs were well-formed, with the exception of the absence of the patella. The knees had a certain amount of permanent flexion, and the patient walked with them at nearly a right angle with the thigh. The feet were turned inward, so that if the leg was extended and he was standing upright, pressure would be made directly on the outer side of the foot. In the position in which he commonly moved, with the body bent upon the thighs, and the legs flexed upon the thighs he brought the soles of the feet in this position nearly parallel with a flat surface.

